# Interleukin-17 vs. Interleukin-23 Inhibitors in Pustular and Erythrodermic Psoriasis: A Retrospective, Multicentre Cohort Study

**DOI:** 10.3390/jcm12041662

**Published:** 2023-02-19

**Authors:** Gianluca Avallone, Carlo Alberto Maronese, Giulia Murgia, Carlo Giovanni Carrera, Luca Mastorino, Gabriele Roccuzzo, Paolo Dapavo, Silvia Alberti-Violetti, Pietro Quaglino, Simone Ribero, Angelo Valerio Marzano

**Affiliations:** 1 Dermatology Clinic, Department of Medical Sciences, University of Turin, 10124 Turin, Italy; 2 Dermatology Unit, Department of Internal Medicine, Fondazione IRCCS Ca’ Granda Ospedale Maggiore Policlinico, 20122 Milan, Italy; 3 Department of Pathophysiology and Transplantation, University of Milan, 20122 Milan, Italy

**Keywords:** pustular psoriasis, erythrodermic psoriasis, interleukin-17 inhibitors, interleukin-23 inhibitors

## Abstract

Pustular and erythrodermic psoriasis are rare and difficult-to-treat conditions. It has recently been shown that interleukin (IL)-17 inhibitors can be very effective among patients with these forms of psoriasis; however, the potential of IL-23 inhibitors is largely unknown. The aim of this multicentre, retrospective study was to compare the safety, effectiveness, and drug survival of IL-17 and IL-23 inhibitors among patients affected by these rare forms of psoriasis. The study involved 27 patients with erythrodermic psoriasis and 59 with pustular psoriasis (36 with generalised pustular psoriasis and 23 with palmoplantar pustular psoriasis) treated with an IL-17 or IL-23 inhibitor. The effectiveness of the two drug classes was assessed using the disease-specific Psoriasis Area Severity Index (PASI) and the Investigator Global Assessment, which were evaluated at different time points. There was a consistent trend towards a higher rate of PASI 100 responses in the patients treated with IL-17 inhibitors compared with those treated with IL-23 inhibitors, and the other efficacy outcomes showed a similar trend. There was no significant between-drug class difference in efficacy at any of the time points in the erythrodermic psoriasis cohort, whereas PASI 90 and PASI 100 response rates were significantly higher among the pustular psoriasis patients receiving IL-17 inhibitors at week 12 (IL-23 19% vs. IL-17 54% and IL-23 6% vs. IL-17 40%, respectively) and the percentage of responders to IL-17 inhibition was significantly higher at week 24 (IL-23 25% vs. IL-17 74%). In conclusion, it is therefore reasonable to assume that IL-17 and IL-23 inhibitors are both effective when treating pustular and erythrodermic psoriasis.

Pustular (PP) and erythrodermic psoriasis (EP) are rare and difficult-to-treat conditions [1]. It has recently been shown that they can be very effectively treated using interleukin(IL)-17 inhibitors [2]; however, little is known about the potential of IL-23 inhibitors because no real-life comparative studies are currently available. The aim of this retrospective, multicentre cohort study was to compare the safety, effectiveness, and drug survival of IL-17 and IL-23 inhibitors among 27 EP and 59 PP patients (36 with generalised pustular psoriasis [GPP] and 23 with palmoplantar pustular psoriasis [PPP]) attending two Italian tertiary referral centres (the Dermatology Clinic at the Turin University Hospital and the Dermatology Unit at Fondazione IRCCS Ca’ Granda Ospedale Maggiore Policlinico of Milan) between October 2019 and July 2022. This study was carried out in accordance with the principles of the Declaration of Helsinki. All patients provided written informed consent for study participation. The study was approved by the ethics committee of Turin University hospital (IT10771180014 SS-Dermo20).

The study inclusion criteria were: (1) an age of ≥18 years; (2) biopsy-confirmed EP (i.e., psoriasis affecting ≥80% of the body surface area [BSA] with inflammatory erythema at baseline) or PP diagnosed based on the 2017 European Consensus Statement [3]; and (3) treatment with an IL-17 or IL-23 inhibitor. Patients with a nondefinitive diagnosis and those aged <18 years were excluded from the study.

Disease severity data were collected at baseline and after 12, 24 and 48 weeks, and drug survival and safety data were available until week 52. The EP group was assessed using the Psoriasis Area Severity Index (PASI) and 5-point Investigator Global Assessment (IGA), and the PP group was assessed using the Generalised Pustular Psoriasis Area and Severity Index (GPPASI), the 5-point Generalised Pustular Psoriasis Investigator Global Assessment (GPPGA), the 5-point Palmoplantar Pustular Psoriasis Investigator Global Assessment (PPPGA), and the Palmoplantar Pustular Psoriasis Area Severity Index (PPPASI). EP treatment responders were retrospectively defined as patients with a PASI90 or PASI100 response, an IGA score of 0–1, or a 2-point decrease from baseline in the IGA score. PP treatment responders were those with a GPPASI90/PPPASI90 or GPPASI90/PPPASI100 response, a GPPGA/PPPGA score of 0–1, or a 2-point decrease from the baseline GPPGA/PPPGA score. Data concerning treatment switches, discontinuations, and side effects were also collected.

Continuous variables are expressed as median values and interquartile ranges (IQR), and categorical variables as absolute numbers and percentages. Fisher’s exact test and the Kruskal–Wallis test were used to assess differences in the frequencies of the categorical variables and the distribution of continuous variables, respectively. All analyses were performed using SAS^®^ statistical analysis software (version 9.4, SAS Institute Inc., Cary, NC, USA).

Table 1 shows the patients’ demographic and clinical features at baseline and during treatment (none of the EP patients was treated with tildrakizumab). There were no differences between the two treatment groups in either cohort, except for higher GPPASI/PPPASI scores and lower frequencies of psoriatic arthritis among the PP patients treated with anti-IL-17 agents.

Although there was no statistically significant difference between the treatment groups in the EP cohort, there was a consistent trend towards a higher rate of disease-specific PASI 100 responses among the patients treated with IL-17 inhibitors. The other efficacy outcomes showed a similar trend, with the notable exception of a higher proportion of patients receiving IL-23 inhibitors than IL-17 inhibitors who achieved a specific PASI 90 response at week 12 in the EP cohort (IL-23 50% vs. IL-17 39%).

A similar trend towards a higher rate of disease-specific PASI 100 responses among the patients treated with IL-17 inhibitors was also documented in the PP group, with differences in the proportions of PASI 90 and PASI 100 responders at week 12 reaching statistical significance (*p* = 0.011 and *p* = 0.020, respectively). Although a higher proportion of patients using IL-23 inhibitors reached respondent status at week 12 (IL-23 50% vs. IL-17 30%), this difference was not statistically significant.

Among the GPP patients, IL-17 inhibition led to higher rates of GPPASI 90 (IL-17 70% vs. IL-23 22%, *p* = 0.022) and GPPASI 100 responses at week 12 (IL-17 56% vs. IL-23 11%, *p* = 0.019) and GPPASI 90 responses at week 24 (IL-17 63% vs. IL-23 56%, *p* = 0.999). The trend in general responsiveness rates was similar (IL-17 82% vs. IL-23 44% at week 12, and IL-17 85% vs. IL-23 22% at week 24); however, the differences were not statistically significant.

In the PPP cohort, IL-17 inhibitor agents led to better efficacy outcomes than IL-23 inhibitors, albeit non-significantly (Table 2).

IL-17 inhibitors had a good safety profile, with nonfatal serious adverse events occurring in 5/43 PP patients (12%) and 1/22 EP patients (5%), and IL-23 inhibitors were associated with only 1/16 (6%) low-grade adverse events (in the PP cohort). Finally, median drug survival was 52 weeks in both disease groups, regardless of treatment. Figure 1, Figure 2, Figure 3 and Figure 4 depict the results for each cohort in the study.

To the best of our knowledge, this is the first study comparing the use of IL-17 and IL-23 inhibitors among patients with PP or EP and utilizes the largest published sample of EP and PP patients treated with anti-IL-23 agents in a real-life setting. Data concerning the effects of IL-23 inhibitors on these subtypes of psoriasis have previously been limited to a few clinical trials, case reports, and case series [4,5,6,7,8]. Cytokines such as IL-17A, IL-23, tumour necrosis factor alpha, and their interferons are overexpressed in GPP and chronic plaque psoriasis lesions [9]. It has been shown that IL-36 is a major driver of the pathogenesis of PP and the recent EFFISAYIL 1 trial has found that a new drug targeting IL-36 (spesolimab) acts selectively and rapidly among patients with GPP. It is therefore likely to be available for use in Europe in the not-too-distant future [10]. One recent fluorescence-activated cell sorting study of the skin lesions associated with these diseases has shown that the proportion of IL-17A-expressing lymphocytes is lower in GPP than in chronic plaque psoriasis, whereas the percentages of IL-23-producing cells are comparable [8]. However, Bissonnette et al. suggest that IL-17A may play an important role in the pathogenesis of PPP, whereas IL-23 expression in PPP may not differ significantly from that observed in other forms of psoriasis [11]. These observations may at least partially explain the clinical improvements observed in our study and suggest that IL-23 may be a possible therapeutic target among patients with either EP or PP.

Our findings suggest that IL-17 and IL-23 inhibitors are similarly effective in treating EP and confirm the effectiveness of the direct inhibition of IL-17 reported by other authors [12,13,14,15,16]. In the case of PP, IL-17 inhibitors tended to be more effective only at the earlier time points and not in a consistent way.

Our study has some clinical and methodological limitations. First, our aggregation of data regarding different drugs belonging to the same biological class may have led to some minor distortions; however, our main objective was to make the most of our sample size and answer a more general clinical question that may apply to a wider range of patients in clinical practice. Second, although short-term efficacy is clearly important in the case of such a severely debilitating disease, it could not be investigated because data regarding the first 12 weeks of drug administration were rarely available (an intrinsic limitation of many retrospective studies). Finally, given the relatively small sample size and retrospective nature of the study, it is not possible to draw any firm conclusions concerning the comparative effectiveness and safety of IL-17 and IL-23 inhibitors.

In conclusion, the results of our study of IL-17 and IL-23 inhibitors in the treatment of EP and PP patients attending two tertiary referral centres indicate that if one of the classes is contraindicated or poorly tolerated it can be expected that the other will be similarly beneficial. However, further studies of the efficacy and safety of biologics for the treatment of EP and PP are needed to establish the optimal approach to these rare forms of psoriasis.

## Figures and Tables

**Figure 1:**
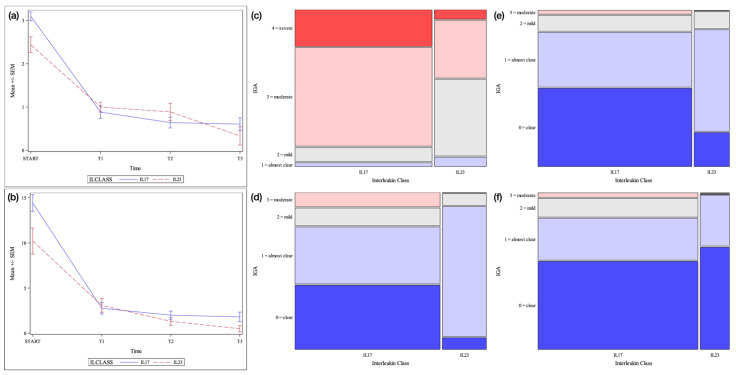
Pustular psoriasis cohort. Legend. (**a**) = Mean +/− SEM IGA score by time point and interleukin class. SEM = Standard error of the mean (**b**) = Mean +/− SEM disease-specific PASI score by time point and interleukin class (**c**) = Mosaic plot of IGA scores by interleukin class: baseline (**d**) = Mosaic plot of IGA scores by interleukin class: week 12 (**e**) = Mosaic plot of IGA scores by interleukin class: week 24 (**f**) = Mosaic plot of IGA scores by interleukin class: week 48.

**Figure 2:**
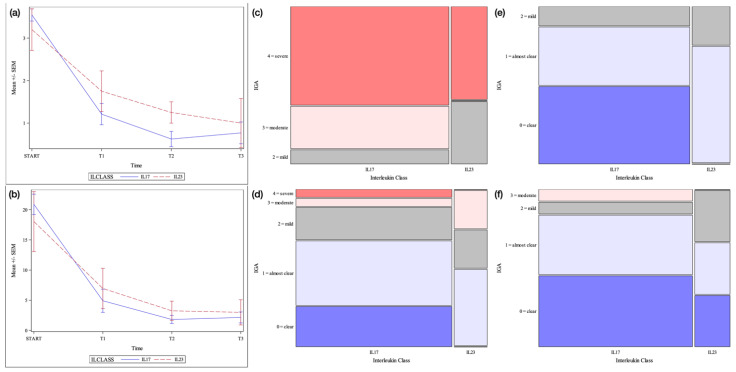
Erythrodermic psoriasis cohort. Legend: (**a**) = Mean +/− SEM IGA score by time point and interleukin class. SEM = Standard error of the mean (**b**) = Mean +/− SEM PASI score by time point and interleukin class (**c**) = Mosaic plot of IGA scores by interleukin class: baseline (**d**) = Mosaic plot of IGA scores by interleukin class: week 12 (**e**) = Mosaic plot of IGA scores by interleukin class: week 24 (**f**) = Mosaic plot of IGA scores by interleukin class: week 48.

**Figure 3:**
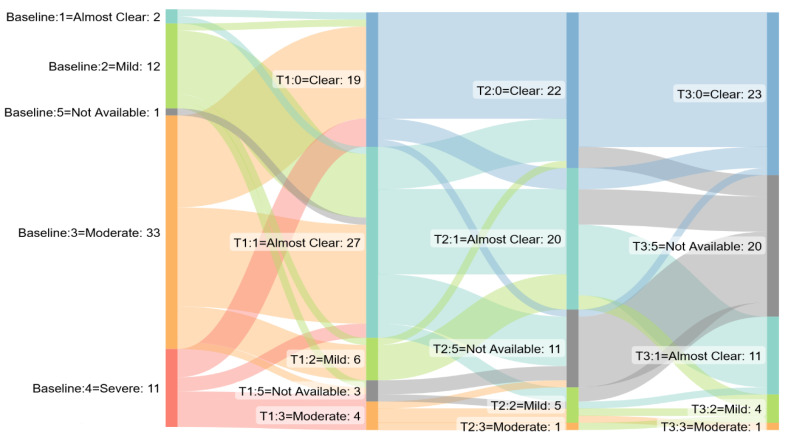
Sankey diagram: GPPGA/PPPGA score by time point in pustular psoriasis cohort. T1 = week 12, T2 week 24, T3 week 48.

**Figure 4:**
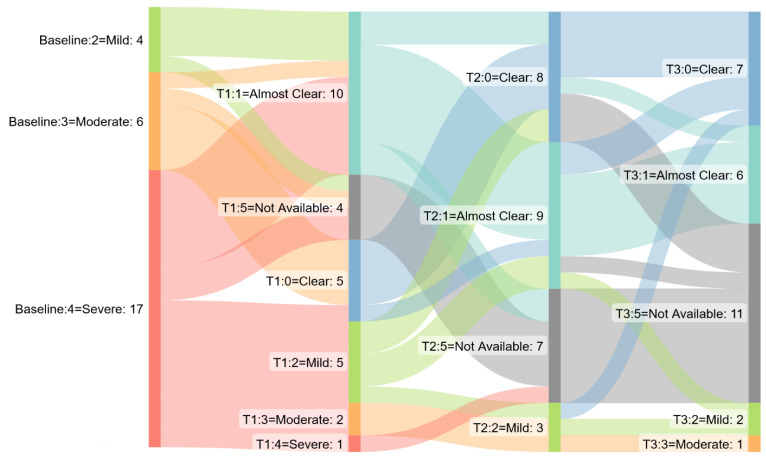
Sankey diagram: IGA score by time point in erythrodermic psoriasis cohort. T1 = week 12, T2 week 24, T3 week 48.

**Table 1:** Baseline and outcome variables by disease and treatment group.

		Erythrodermic Psoriasis			Pustular Psoriasis		
Variable		IL23 (N = 6)	IL17 (N = 23)	*p*-Value	IL23 (N = 16)	IL17 (N = 43)	*p*-Value
Sex	Male	3 (50%)	14 (61%)	1	5 (31%)	13 (30%)	0.499
	Female	2 (33%)	8 (35%)		7 (44%)	30 (70%)	
Height (cm)	Median [Q1–Q3]	180.00 [180.00–180.00]	173.00 [165.00–175.00]	0.170	160.00 [160.00–170.00]	166.00 [160.00–172.00]	0.183
Weight (kg)	Median [Q1–Q3]	85.00 [75.00–85.00]	75.00 [70.00–85.00]	0.534	65.00 [61.00–73.00]	75.00 [65.00–85.00]	0.069
Age at diagnosis	Median [Q1–Q3]	22.00 [20.00–40.00]	33.50 [20.50–52.50]	0.683	39.00 [25.00–48.00]	42.50 [22.00–53.00]	0.432
Education	Primary school	2 (33%)	3 (13%)	0.228	0 (0%)	2 (5%)	0.817
	Middle school	0 (0%)	7 (30%)		3 (19%)	11 (26%)	
	High school	2 (33%)	9 (39%)		8 (50%)	21 (49%)	
	Higher education	1 (17%)	2 (9%)		3 (19%)	5 (12%)	
Smoke	Never	2 (33%)	9 (39%)	0.331	5 (31%)	13 (30%)	0.860
	Former	2 (33%)	3 (13%)		5 (31%)	18 (42%)	
PsA	Yes	2 (33%)	18 (78%)	0.091	13 (81%)	22 (51%)	0.043
	No	3 (50%)	4 (17%)		3 (19%)	21 (49%)	
Comorbidities	Yes	0 (0%)	7 (30%)	0.283	8 (50%)	27 (63%)	0.39
	No	5 (83%)	15 (65%)		8 (50%)	16 (37%)	
CV comorbidities	Yes	3 (50%)	12 (52%)	1	5 (31%)	18 (42%)	0.444
	No	2 (33%)	9 (39%)		3 (19%)	24 (56%)	
Diabetes	Yes	0 (0%)	2 (9%)	1	2 (13%)	4 (9%)	0.325
	No	5 (83%)	19 (83%)		8 (50%)	38 (88%)	
Naive Biologic	Yes	2 (33%)	15 (65%)	0.326	8 (50%)	24 (56%)	0.769
	No	3 (50%)	7 (30%)		8 (50%)	18 (42%)	
Obesity	Yes	0 (0%)	3 (13%)	1	1 (6%)	11 (26%)	0.152
	No	5 (83%)	19 (83%)		14 (88%)	31 (72%)	
Baseline DLQI	Median [Q1–Q3]	18.50 [18.00–19.00]	30.00 [18.00–30.00]	0.246	27.00 [24.00–28.00]	28.00 [26.00–30.00]	0.350
Baseline PASI	Median [Q1–Q3]	20.00 [8.00–27.00]	22.00 [14.00–27.00]	0.683	8.20 [6.00–13.50]	15.00 [10.00–19.00]	0.009
Previous therapies	Acitretin	3 (50%)	6 (26%)	0.915	5 (31%)	18 (42%)	0.750
	Apremilast	0 (0%)	0 (0%)		2 (13%)	8 (19%)	
	Cyclosporine	4 (67%)	11 (48%)		3 (19%)	20 (47%)	
	MTX	2 (33%)	9 (39%)		11 (69%)	28 (65%)	
	Phototherapy	1 (17%)	4 (17%)		1 (6%)	5 (12%)	
	Oral steroids	0 (0%)	0 (0%)		0 (0%)	4 (9%)	
Administered drug ^§^	Risankizumab	3 (50%)	-	-	8 (50%)	-	-
	Guselkumab	2 (33%)	-		4 (25%)	-	
	Tildrakizumab	0 (0%)	-		4 (25%)	-	
	Secukinumab	-	11 (48%)		-	17 (40%)	
	Brodalumab	-	7 (30%)		-	14 (33%)	
	Ixekizumab	-	4 (17%)		-	12 (28%)	
Is patient respondent? *							
at week 12	Yes	1 (17%)	11(48%)	0.317	8 (50%)	13 (30%)	0.055
	No	3 (50%)	8 (35%)		5 (31%)	30 (70%)	
at week 24	Yes	2 (33%)	13(57%)	0.249	4 (25%)	32 (74%)	0.032
	No	2 (33%)	3 (13%)		5 (31%)	7 (16%)	
at week 48	Yes	2 (33%)	10(44%)	0.999	5 (31%)	27 (63%)	1
	No	1 (17%)	3 (13%)		1 (6%)	6 (14%)	
PASI 100 ^#^							
at week 12	Yes	0 (0%)	7 (30%)	0.295	1 (6%)	17 (40%)	0.011
	No	4 (67%)	14(70%)		15(94%)	23 (54%)	
at week 24	Yes	0 (0%)	11(48%)	0.093	3 (19%)	20 (47%)	0.066
	No	4 (67%)	8 (35%)		11 (69%)	19 (44%)	
at week 48	Yes	1 (17%)	11 (48%)	0.553	4 (25%)	20 (47%)	1
	No	2 (33%)	7 (30%)		3 (19%)	15 (35%)	
PASI 90 ^#^							
at week 12	Yes	3 (50%)	9 (39%)	0.59	3 (19%)	23 (54%)	0.02
	No	1 (17%)	11(48%)		13 (81%)	20 (47%)	
at week 24	Yes	2 (33%)	15 (65%)	0.271	7 (44%)	23 (54%)	0.757
	No	2 (33%)	4 (17%)		7 (44%)	17 (40%)	
at week 48	Yes	1 (17%)	14 (61%)	0.184	6 (38%)	24 (56%)	0.651
	No	2 (33%)	4 (17%)		1 (6%)	11 (26%)	
Switch	Yes	0 (0%)	7 (30%)	0.283	2 (13%)	8 (19%)	0.713
	No	5 (83%)	15 (65%)		14 (88%)	35 (81%)	
Discontinuation	Yes	1 (17%)	18 (78%)	0.636	2 (13%)	8 (19%)	0.713
	No	4 (67%)	14 (61%)		14 (88%)	35 (81%)	
Reason for discontinuation	Other	0 (0%)	1 (4%)	0.786	2 (13%)	3 (7%)	0.444
	Collateral effect	0 (0%)	1 (4%)		0 (0%)	0 (0%)	
	Primary inefficacy	1 (17%)	3 (13%)		0 (0%)	1 (2%)	
	Loss of efficacy	0 (0%)	3 (13%)		0 (0%)	3 (7%)	
Adverse events		0 (0%)	1 (4%)		1 (6%)	5 (12%)	
	Acne	0 (0%)	1 (4%)		0 (0%)	0 (0%)	
	Asthenia	0 (0%)	0 (0%)		0 (0%)	1 (2%)	
	Breast dermatitis	0 (0%)	0 (0%)		1 (6%)	0 (0%)	
	Pustulation on the back	0 (0%)	0 (0%)		0 (0%)	1 (2%)	
	Hand/foot pain after first administration	0 (0%)	0 (0%)		0 (0%)	1 (2%)	
	Increased hunger in the days following the injection	0 (0%)	0 (0%)		0 (0%)	1 (2%)	
	Injection site reaction	0 (0%)	0 (0%)		0 (0%)	1 (2%)	
Drug survival	Median [Q1-Q3]	52.00 [33.05–52.00]	52.00 [14.09–52.00]	0.616	52.00 [52.00–52.00]	52.00 [52.00–52.00]	0.602

PsA: psoriatic arthritis; IL-17: Interleukin-17; IL-23: Interleukin-23; CV: cardiovascular; DLQI = Dermatology Life Quality Index; MTX: Methotrexate; PASI: Psoriasis Area Severity Index; Q1: quartile 1; Q3: quartile 3; T1: week 12; T2: week 24; T3: week 48 ^§^ all of the subjects received the standard dosing schedule of secukinumab, brodalumab, ixekizumab, risankizumab, tildrakizumab, or risankizumab * Patients considered responders if their IGA score was 0 or 1 or at least two points lower than at baseline ^#^ GPPASI in the case of GPP patients, PPPASI in the case of PPP patients.

**Table 2:** Baseline and outcome variables in the PP cohort by variant and treatment group.

		Generalised PP			Palmoplantar PP		
Variable		IL23 (N = 9)	IL17 (N = 27)	*p*-Value	IL23 (N = 7)	IL17 (N = 16)	*p*-Value
Sex	Male	5 (56%)	10 (37%)	0.443	0 (0%)	3 (19%)	1
	Female	4 (44%)	17 (63%)		3 (43%)	13 (81%)	
Height (cm%)	Median [Q1–Q3]	162.50 [160.00–176.00]	165.00 [160.00–172.00]	0.882	160.00 [155.00–164.00]	167.00 [160.00–170.00]	0.082
Weight (kg%)	Median [Q1–Q3]	67.00 [58.00–78.50]	79.00 [70.00–86.00]	0.107	65.00 [63.00–73.00]	71.50 [64.00–79.00]	0.503
Age at diagnosis	Median [Q1–Q3]	32.00 [20.00–48.00]	39.00 [20.00–52.00]	0.597	45.00 [35.00–50.00]	49.50 [39.00–55.00]	0.3
Education	Primary school	0 (0%)	2 (7%)	0.747	0 (0%)	0 (0%)	0.499
	Middle school	3 (33%)	8 (30%)		0 (0%)	3 (19%)	
	High school	2 (22%)	1 (4%)		1 (14%)	1 (6%)	
	Higher education	2 (22%)	4 (15%)		6 (86%)	10 (63%)	
Smoke	Never	4 (44%)	7 (26%)	0.26	1 (14%)	6 (38%)	0.549
	Former	2 (22%)	14 (52%)		3 (43%)	4 (25%)	
	Current	1 (11%)	5 (19%)		3 (43%)	6 (38%)	
PsA	Yes	0 (0%)	13 (48%)	0.014	3 (43%)	8 (50%)	1
	No	9 (100%)	14 (52%)		4 (57%)	8 (50%)	
Comorbidities	Yes	3 (33%)	18 (67%)	0.122	5 (71%)	9 (56%)	0.657
	No	6 (67%)	9 (33%)		2 (29%)	7 (44%)	
CV comorbidities	Yes	2 (22%)	14 (52%)	0.654	2 (29%)	6 (38%)	0.546
	No	3 (33%)	12 (44%)		1 (14%)	10 (63%)	
Diabetes	Yes	1 (11%)	4 (15%)	0.999	1 (14%)	0 (0%)	0.2
	No	5 (56%)	22 (81%)		3 (43%)	16 (100%)	
Naive Biologic	Yes	7 (78%)	14 (52%)	0.262	1 (14%)	10 (63%)	0.069
	No	2 (22%)	12 (44%)		6 (86%)	6 (38%)	
Obesity	Yes	0 (0%)	10 (37%)	0.072	1 (14%)	1 (6%)	0.526
	No	8 (89%)	16 (59%)		6 (86%)	15 (94%)	
Baseline DLQI	Median [Q1–Q3]	25.50 [19.50–28.50]	28.00 [26.00–30.00]	0.292	28.00 [28.00–28.00]	28.00 [25.00–30.00]	0.868
Baseline PASI	Median [Q1–Q3]	12.00 [7.00–16.00]	16.00 [12.00–20.00]	0.057	7.00 [6.00–10.00]	10.00 [8.00–15.50]	0.119
Previous therapies	Acitretin	2 (22%)	10 (37%)	0.651	3 (43%)	8 (50%)	0.982
	Apremilast	1 (11%)	6 (22%)		1 (14%)	2 (13%)	
	Cyclosporine	1 (11%)	12 (44%)		2 (29%)	8 (50%)	
	MTX	7 (78%)	17 (63%)		4 (57%)	11 (69%)	
	Phototherapy	1 (11%)	4 (15%)		0 (0%)	1 (6%)	
	Oral steroids	0 (0%)	4 (15%)		0 (0%)	0 (0%)	
Administered drug	Risankizumab	5 (56%)	-	-	3 (43%)	-	-
	Guselkumab	0 (0%)	-		4 (57%)	-	
	Tildrakizumab	4 (44%)	-		0 (0%)	-	
	Secukinumab	-	9 (33%)		-	8 (50%)	
	Brodalumab	-	11 (41%)		-	3 (19%)	
	Ixekizumab	-	7 (26%)		-	5 (31%)	
Is patient respondent? *							
at week 12	Yes	4 (44%)	22 (82%)	0.162	1 (14%)	8 (50%)	0.338
	No	4 (44%)	5 (19%)		4 (57%)	8 (50%)	
at week 24	Yes	2 (22%)	23 (85%)	0.119	2 (29%)	9 (56%)	0.326
	No	2 (22%)	3 (11%)		3 (43%)	4 (25%)	
at week 48	Yes	4 (44%)	17 (63%)	1	1 (14%)	10 (63%)	1
	No	1 (11%)	5 (19%)		0 (0%)	1 (6%)	
PASI 100 ^#^							
at week 12	Yes	1 (11%)	15 (56%)	0.022	0 (0%)	2 (13%)	0.533
	No	8 (89%)	11 (41%)		7 (100%)	12 (75%)	
at week 24	Yes	6 (67%)	11 (41%)	0.225	1 (14%)	6 (38%)	0.354
	No	2 (22%)	14 (52%)		5 (71%)	8 (50%)	
at week 48	Yes	4 (44%)	13 (48%)	0.673	0 (0%)	7 (44%)	0.417
	No	2 (22%)	11 (41%)		1 (14%)	4 (25%)	
PASI 90 ^#^							
at week 12	Yes	2 (22%)	19 (70%)	0.019	1 (14%)	4 (25%)	1
	No	7 (78%)	8 (30%)		6 (86%)	12 (75%)	
at week 24	Yes	5 (56%)	17 (63%)	1	2 (29%)	6 (38%)	1
	No	3 (33%)	9 (33.3%)		4 (57%)	8 (50%)	
at week 48	Yes	5 (56%)	16 (59%)	0.637	1 (14%)	8 (50%)	1
	No	1 (11%)	8 (30%)		0 (0%)	3 (19%)	
Switch	Yes	0 (0%)	5 (19%)	0.302	2 (29%)	3 (19%)	0.621
	No	9 (100%)	22 (81%)		5 (71%)	13 (81%)	
Discontinuation	Yes	0 (0%)	6 (22%)	0.303	2 (29%)	2 (13%)	0.557
	No	9 (100%)	21 (78%)		5 (71%)	14 (88%)	
Reason for discontinuation	Other	0 (0%)	3 (11%)		2 (29%)	0 (0%)	
	Collateral effect	0 (0%)	0 (0%)		0 (0%)	0 (0%)	
	Primary inefficacy	0 (0%)	0 (0%)		0 (0%)	1 (6%)	
	Loss of efficacy	0 (0%)	2 (7%)		0 (0%)	1 (6%)	
Adverse events		0 (0%)	5 (19%)		1 (14%)	0 (0%)	
	Asthenia	0 (0%)	1 (4%)		0 (0%)	0 (0%)	
	Breast dermatitis	0 (0%)	0 (0%)		1 (14%)	0 (0%)	
	Pustulation on the back	0 (0%)	1 (4%)		0 (0%)	0 (0%)	
	Hand/foot pain after first administration	0 (0%)	1 (4%)		0 (0%)	0 (0%)	
	Increased hunger in the days following the injection	0 (0%)	1 (4%)		0 (0%)	0 (0%)	
	Injection site reaction	0 (0%)	1 (4%)		0 (0%)	0 (0%)	
Drug survival	Median [Q1–Q3]	52.00 [52.00–52.00]	52.00 [52.00–52.00]	0.129	52.00 [16.82–52.00]	52.00 [52.00–52.00]	0.479

PsA: psoriatic arthritis; IL-17: Interleukin-17; IL-23: Interleukin-23; CV: cardiovascular; DLQI = Dermatology Life Quality Index; MTX: Methotrexate; PASI = Psoriasis Area Severity Index; Q1: quartile 1; Q3: quartile 3; T1: week 12; T2: week 24; T3: week 48 * Patients considered responders if their IGA score was 0 or 1 or at least two points lower than at baseline ^#^ EP treatment responders were retrospectively defined as patients with a PASI90 or PASI100 response, an IGA score of 0–1, or a 2-point decrease from the baseline IGA score. PP treatment responders were those with a GPPASI90/PPPASI90 or GPPASI90/PPPASI100 response, a GPPGA/PPPGA score of 0–1, or a 2-point decrease from the baseline GPPGA/PPPGA score.

## Data Availability

The data that support the findings of this study are available from the corresponding author.
